# Function and Mechanism of Trimetazidine in Myocardial Infarction-Induced Myocardial Energy Metabolism Disorder Through the SIRT1–AMPK Pathway

**DOI:** 10.3389/fphys.2021.645041

**Published:** 2021-06-17

**Authors:** Xiu-ying Luo, Ze Zhong, Ai-guo Chong, Wei-wei Zhang, Xin-dong Wu

**Affiliations:** Department of Cardiology, The Second Affiliated Hospital (Jiande Branch), Zhejiang University School of Medicine, Hangzhou, China

**Keywords:** myocardial energy metabolism disorder, trimetazidine, SIRT1-AMPK pathway, myocardial infarction, apoptosis

## Abstract

Myocardial energy metabolism (MEM) is an important factor of myocardial injury. Trimetazidine (TMZ) provides protection against myocardial ischemia/reperfusion injury. The current study set out to evaluate the effect and mechanism of TMZ on MEM disorder induced by myocardial infarction (MI). Firstly, a MI mouse model was established by coronary artery ligation, which was then treated with different concentrations of TMZ (5, 10, and 20 mg kg^–1^ day^–1^). The results suggested that TMZ reduced the heart/weight ratio in a concentration-dependent manner. TMZ also reduced the levels of Bax and cleaved caspase-3 and promoted Bcl-2 expression. In addition, TMZ augmented adenosine triphosphate (ATP) production and superoxide dismutase (SOD) activity induced by MI and decreased the levels of lipid peroxide (LPO), free fatty acids (FFA), and nitric oxide (NO) in a concentration-dependent manner (all *P* < 0.05). Furthermore, an H_2_O_2_-induced cell injury model was established and treated with different concentrations of TMZ (1, 5, and 10 μM). The results showed that SIRT1 overexpression promoted ATP production and reactive oxygen species (ROS) activity and reduced the levels of LPO, FFA, and NO in H9C2 cardiomyocytes treated with H_2_O_2_ and TMZ. Silencing SIRT1 suppressed ATP production and ROS activity and increased the levels of LPO, FFA, and NO (all *P* < 0.05). TMZ activated the SIRT1–AMPK pathway by increasing SIRT1 expression and AMPK phosphorylation. In conclusion, TMZ inhibited MI-induced myocardial apoptosis and MEM disorder by activating the SIRT1–AMPK pathway.

## Introduction

Myocardial infarction (MI) persists as a vital cause of high mortality and morbidity worldwide (Bajaj et al., 2015). MI is characterized by the formation of plaques in the internal walls of arteries, leading to restricted blood flow to the heart and injuring the heart muscles due to depletion of the oxygen supply (Lu et al., 2015). Currently, timely coordinated myocardial reperfusion is regarded as the gold standard for MI treatment; however, the reperfusion process can induce cardiomyocyte death (Hausenloy and Yellon, 2013). Cardiomyocyte death, typified by necrosis, apoptosis, and autophagy, can be triggered due to dysregulated myocardial energy metabolism (MEM) (Tao et al., 2015). Due to the dynamic nature and significance of energy metabolism, the heart’s ability to metabolize a broad spectrum of energy substrates to incur with the extensive requirements is widely known (Lopaschuk and Ussher, 2016). MEM alterations can manifest both as a deficit in energy production by the heart and as a decrease in cardiac efficiency, which facilitates the progression of heart failure (Mori et al., 2013). Meanwhile, the process of apoptosis is regarded as the trivial cause of cardiomyocyte fate and myocardial remodeling after MI (Foglio et al., 2019). Inhibiting apoptosis and improving the energy metabolism have emerged as protective strategies against MI (Lim and Lee, 2017). Therefore, further investigations are warranted to explore the mechanisms of inhibiting apoptosis and improving energy metabolism for the management of MI.

Trimetazidine (TMZ) is a metabolic anti-ischemic agent that can shift the energy substrate metabolism and enhance glucose metabolism (Kallistratos et al., 2019). Clinically, TMZ is administered as treatment for angina pectoris and heart failure (Shu et al., 2020; Amoedo et al., 2021). Existing evidence has demonstrated the participation of TMZ in severe adverse cardiac events, myocardial metabolic remodeling, and percutaneous coronary intervention by the activation of AMPK and PPARα (Ferrari et al., 2020; Li et al., 2020; Zhu et al., 2020). The 3-ketoacyl-CoA thiolase protein, which is associated with energy supply and metabolism, is inhibited by TMZ (Li et al., 2020; Yan et al., 2020). A recent study elicited the ability of TMZ treatment to significantly reduce the MI size in mice (Gong et al., 2018). Moreover, TMZ exhibits an advancing effect on non-alcoholic fatty liver disease and sunitinib-induced cardiotoxicity (Yang et al., 2019; Zhang et al., 2020). AMPK, an evolutionarily conserved energy sensor, principally serves as a regulator of cellular metabolism, while silent information regulator 1 (SIRT1) is a protein deacetylase that regulates the life span extension and gene silencing in yeast (Fulco and Sartorelli, 2008). SIRT1 can function as deacetylating anti-inflammatory and anti-apoptotic molecules to counter cerebral hypoperfusion and ischemia (Hattori and Ihara, 2016). AMPK activation modulates glucose and fatty acid metabolism and apoptosis during ischemia–reperfusion (Qi and Young, 2015). Research supports that the effect of TMZ in macrophages is conducive by normalizing SIRT1/AMPK, while the anti-inflammatory effect of TMZ could be induced by the activation of SIRT1 (Chen et al., 2016). Currently, no report has documented whether the SIRT1–AMPK pathway participates in the regulation of TMZ in MI-induced MEM disorder domestically and abroad, until now. Therefore, in this study, we explored the therapeutic effect of TMZ on MI in an attempt to uncover new theoretical basis for TMZ-mediated myocardial apoptosis and MEM disorder affecting MI.

## Materials and Methods

### Ethics Statement

All animal experiments were conducted in compliance with the guidelines for experimental animal care and use, ARRIVE guidelines and the guidelines of the International Association for the Study of Pain, and were approved by the Animal Care and Use Committee of the Second Affiliated Hospital of Zhejiang University. Adequate measures were taken to minimize animal suffering. All procedures were strictly implemented by the code of ethics.

### Establishment and Grouping of MI Mice

Healthy male C57BL/6 mice (10 weeks old) were provided by Vital River Laboratory Animal Technology Co. Ltd. (Beijing, China). The MI model was established by coronary artery ligation. The percentage of mortality was 10% with a mortality of 10 mice in each group during the experiment. Mice were assigned into nine groups as follows: sham group, MI group, MI + TMZ + L group (MI mice treated with 5 mg kg^–1^ day^–1^ TMZ), MI + TMZ + M group (MI mice treated with 10 mg kg^–1^ day^–1^ TMZ), MI + TMZ + H group (MI mice treated with 20 mg kg^–1^ day^–1^ TMZ), MI + TMZ + oe-NC group (MI mice treated with TMZ and lentivirus overexpression), MI + TMZ + oe-SIRT1 group (MI mice treated with TMZ and SIRT1 overexpression lentivirus), MI + TMZ + sh-NC group (MI mice treated with TMZ and silencing lentivirus), and MI + TMZ + sh-SIRT1 group (MI mice treated with TMZ and SIRT1 knockdown lentivirus). The mice were anesthetized using an intraperitoneal injection of pentobarbital sodium (50 mg/kg) and connected to a respirator (Inspira ASVP, Harvard Apparatus, Holliston, MA, United States) for mechanical ventilation. Next, a left thoracotomy and pericardiotomy were performed. A 7–0 polypropylene suture was used to ligate the left anterior descending (LAD) coronary artery at 2–3 mm from the left atrial appendage. Subsequently, 10 μl [1 × 10^8^ transduction unit (TU)/ml] of lentivirus (oe-SIRT1, sh-SIRT1, and corresponding controls) was injected into the myocardium around the ligation area (above, below, left, and down), with 2.5 μl injected in each area. The same surgery was performed to mice in the sham group without coronary artery ligation (Zhang et al., 2020). As for TMZ treatment, mice that survived 12 h after surgery were intraperitoneally injected with normal saline or different concentrations of TMZ (5, 10, and 20 mg kg^–1^ day^–1^) for 7 days (Jain et al., 2011). Mice were euthanized after surgery for further experimentation. Within each group, three mice were reserved for triphenyltetrazolium chloride (TTC) staining, three for histopathological experiment, and the rest were reserved for Western blot and PCR experiments. The body and heart weights of each mice were documented and the heart/weight ratio was calculated (Leung et al., 2021). The lentiviral vectors specifically targeting the sequence of SIRT1 knockdown (sh-SIRT1), scramble shRNA (sh-NC) as a control, and SIRT1 overexpression (oe-SIRT1) and its control (oe-NC) were constructed by GenePharma Co. (Shanghai, China). The lentiviral vectors and the packaging vector were transfected to HEK293T cells using Lipofectamine 3000 (Invitrogen, L3000015, Carlsbad, CA, United States). Lentivirus particles were harvested after 28 h for subsequent experimentation.

### Cardiac Function Evaluation

Mice were anesthetized and ultrasonic electrocardiography was conducted on the 26th day to evaluate cardiac function. Cardiac function was detected using the Vevo^®^ 2100 System equipped with a 30-MHz transducer (FUJIFILM VisualSonics, Inc., Toronto, ON, Canada). Indices such as the left ventricular internal diameter at end-systole (LVIDs), left ventricular internal diameter at end-diastole (LVIDd), left ventricular fraction shortening (LVFS), and left ventricular ejection fraction (LVEF) were all analyzed using the Vevo 2100 software (Yao et al., 2020).

### Triphenyltetrazolium Chloride Staining

Myocardial infarct sizes were measured by TTC staining. Mouse hearts were preserved at –80°C for 5 min. Next, the heart tissue was divided into five to six blocks of 1–2 mm thickness and incubated in 1% TTC solution for 30 min at 37°C in conditions devoid of light. The tissue blocks were fixed using 4% paraformaldehyde solutions for 24 h. A digital camera was adopted to document the observations as images and the Image-Pro Plus software (Image-Pro Plus 6.0, Media Cybernetics, Bethesda, MD, United States) was employed to measure the infarct size. Myocardial infarct size (%) = (sum of infarct areas/whole heart areas) × 100% (Tian et al., 2019).

### Hematoxylin and Eosin Staining

As previously described (Tan et al., 2018), the myocardial tissues previously harvested in the infarct areas were fixed for 24 h with 4% paraformaldehyde and embedded in paraffin. Next, hematoxylin and eosin (HE) staining was performed to analyze modifications in the morphology of the myocardial tissues. Staining was observed under an optical microscope to visualize the degree of heart damage. Five sections from each sample were observed by two pathologists independently in a double-blind manner.

### Terminal Deoxyribonucleotidyl Transferase-Mediated Biotin-16-dUTP Staining

An *in situ* cell death detection kit (Roche Diagnostics GmbH, Mannheim, Germany) was used to determine cardiomyocyte apoptosis in compliance with the provided instructions. Briefly, the tissue sections were fixed in 4% paraformaldehyde and incubated in 20 μg/ml proteinase K for 15 min. The tissue sections were rinsed with phosphate-buffered saline and immersed in the terminal deoxynucleotidyl transferase dUTP nick-end labeling (TUNEL) reaction mixture at 37°C for 1 h in a moist chamber. The slides were immersed in 2 × sodium citrate saline solution to terminate the reaction. Endogenous peroxidase activity was terminated by incubation of the slides in 0.3% hydrogen peroxide. Lastly, streptavidin horseradish peroxidase was combined with the biotinylated nucleotides and the peroxidase activity was observed in each section by applying the stable chromogen, diaminobenzidine. Apoptotic cardiomyocyte nuclei appeared brown whereas normal nuclei appeared blue with hematoxylin staining. Five sections from each myocardial sample were randomly selected and assessed by two independent researchers in a double-blind manner. The percentage of TUNEL-positive nuclei was calculated in each field (Jian et al., 2015).

### Western Blot

The protein content was extracted from the tissues in infarcted areas or cells by the addition of enhanced radioimmunoprecipitation assay (RIPA) lysate (Boster, Wuhan, Hubei, China) with protease inhibitors. A bicinchoninic acid (BCA) Protein Assay Kit (Boster) was used to measure the protein concentrations. Protein content was isolated on 10% SDS-PAGE and then transferred onto polyvinylidene fluoride (PVDF) membranes. Membrane blockade was conducted using 5% bovine serum albumin (BSA) for 2 h to eliminate non-specific binding and incubated overnight at 4°C with the diluted primary anti-rabbit Bax (ab32503, dilution ratio of 1:1,000; Abcam, Cambridge, UK, United States), Bcl-2 (ab194583, dilution ratio of 1:2,000; Abcam), cleaved caspase-3 (ab49822, dilution ratio of 1:500; Abcam), SIRT1 (ab233398, dilution ratio of 1:1,000; Abcam), p-AMPK (4188S, dilution ratio of 1:2,000; Cell Signaling Technology, Shanghai, China), t-AMPK (5831S, dilution ratio of 1:1,000; Cell Signaling Technology), and β-actin (ab8227, dilution ratio of 1:5,000; Abcam). The membranes were rinsed and incubated with the horseradish peroxidase (HRP)-tagged secondary antibody of goat anti-rabbit IgG (ab205718, 1:2,000) for 1 h at room temperature. The membranes were visualized using an enhanced chemiluminescence (ECL) working solution (EMD Millipore, Billerica, MA, United States). Gray level analysis was performed using the Image-Pro Plus 6.0 (Media Cybernetics Inc., Bethesda, MD, United States) with β-actin as an internal control. The experiment was independently conducted three times.

### Detection of Energy Metabolism-Related Indices

Colorimetry was applied to detect the content of adenosine triphosphate (ATP; A095-1-1, Jiancheng Bioengineering Institute, Nanjing, China): specimens were treated based on the provided instructions and then placed at room temperature for 5 min; the absorbance value at a wavelength of 636 nm was detected at 0.5 cm optical path. Nitric oxide (NO; A012-1-2, Jiancheng Bioengineering Institute) content was detected as follows: specimens were treated based on the provided instructions and mixed and placed at room temperature for 10 min; the absorbance value at a wavelength of 550 nm was detected at 0.5 cm optical path. Superoxide dismutase (SOD; K335-100, Beyotime, Shanghai, China) activity was measured: specimens were treated based on the provided instructions and supplemented with 200 ml of the SOD detection solution and incubated at 37°C for 3 min; the absorbance value was detected at a wavelength of 450 nm. The thiobarbituric acid (TBA) method was adopted to detect the myocardial lipids: specimens were treated based on the provided instructions and colorimetric assay was performed at a wavelength of 520 nm and the relative levels of malondialdehyde (MDA) detected. The contents of myocardial lipid peroxide (LPO; A106, Jiancheng Bioengineering Institute) were detected as follows: specimens were treated according to the provided instructions, incubated at 45°C for 60 min, and centrifuged at 4,000×*g* for 10 min; 200 μl supernatants were harvested and transferred into 96-well plates. The absorbance value was detected at a wavelength of 586 nm. The optical density (OD) value in each well was detected. Contents of free fatty acids (FFA; xy-E1085, Xin Yu Biotech Co., Ltd., Shanghai, China) were measured: specimens were treated based on the provided instructions and the absorbance value was detected at s wavelength of 550 nm.

### Cell Culture and Treatments

H9C2 cells were obtained from ATCC (CRL-1446, ATCC, Manassas, VA, United States) and cultured in Dulbecco’s modified Eagle’s medium (DMEM; Gibco, Rockville, MD, United States) with 15% fetal bovine serum (FBS; Gibco, Rockville, MD, United States) and 5% CO_2_ at 37°C. Then, the cells were seeded into six-well plates independently. After 24 h, lentivirus oe-SIRT1, sh-SIRT1, and the corresponding controls were infected into H9C2 cells in the presence of polybrene (10 mg/ml) at 1 × 10^8^ TU/ml. After infection for 48 h, H9C2 cells were pretreated with varied TMZ concentrations (1, 5, and 10 μM) for 12 h and then treated with H_2_O_2_ (100 μM) for 4 h to establish the H9C2 cell injury model (Gong et al., 2018; Shi et al., 2020).

### Flow Cytometry

Apoptosis was measured using Annexin V-FITC/propidium iodide (PI) double staining. The cell concentrations in each group were adjusted to 1 × 10^6^ cells/ml. Then, the cells were fixed with 70% pre-cooled ethanol solution and subjected to overnight incubation at 4°C. A total of 100 μl cell suspension (no less than 10^6^ cells/ml) was resuspended in 200 μl binding buffer and stained using 10 μl Annexin V-FITC and 5 μl PI at room temperature for 15 min in conditions devoid of light. Flow cytometric analysis for the determination the apoptosis was performed on BD FACS Canto II (Biosciences, San Jose, CA, United States) after the addition of 300 μl binding buffer (Chen et al., 2018).

### Statistical Analysis

Data were analyzed with SPSS 21.0 software (IBM Corp., Armonk, NY, United States). Data were presented as the mean ± standard deviation (SD). The normal distribution and variance homogeneity were measured first. One-way analysis of variance (ANOVA) was applied to the results in compliance with normal distribution and variance homogeneity, followed by Tukey’s multiple comparisons test. Otherwise, the rank-sum test was applied. A value of *P* < 0.05 was considered statistically significant.

## Results

### Trimetazidine Inhibited MI-Induced Myocardial Apoptosis

**FIGURE 1:**
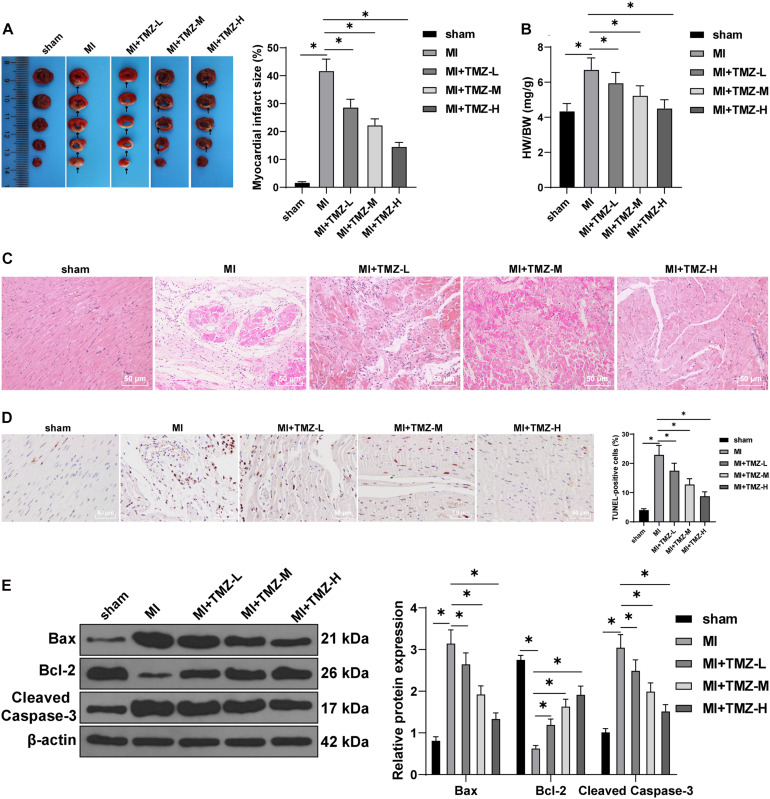
Trimetazidine (TMZ) inhibited myocardial infarction (MI)-induced myocardial apoptosis. **(A)** MI was detected by triphenyltetrazolium chloride (TTC) staining. *Areas in white* represent the MI region. **(B)** Heart weight was measured and the heart/weight ratio calculated. **(C)** Pathological changes of the myocardial tissue were detected by hematoxylin–eosin (HE) staining. **(D)** Myocardial apoptosis was detected by transferase-mediated biotin-16-dUTP (TUNEL) staining. **(E)** Western blot analysis of Bax, Bcl-2, and cleaved caspase-3 (*N* = 3 per group). Detection was repeated three times. All data were expressed as the mean ± standard deviation. Data were analyzed using one-way one-way analysis of variance (ANOVA), followed by Tukey’s multiple comparisons test. *P < 0.05.

An MI mouse model was established to investigate the regulatory function of TMZ on MI. TTC staining was conducted to precisely determine the degree of MI. Ultrasonic electrocardiography showed that LVIDd and LVIDs were increased in MI mice, while LVEF and FS were decreased. MI mice were treated with variable concentrations of TMZ and cardiac function was retrieved after TMZ treatment in a concentration-dependent manner (all *P* < 0.05; Supplementary Table 1). The infarct sizes of the model group were significantly increased relative to the sham group (Figure 1A). Mice were treated with TMZ at low (TMZ-L), moderate (TMZ-M), and high (TMZ-H) concentrations. The TTC staining results showed that TMZ significantly reduced the infarct size in a TMZ concentration-dependent manner (all *P* < 0.05; Figure 1B). Meanwhile, our findings revealed that the heart/weight ratios of MI mice were significantly higher compared to the sham-operated mice, and TMZ treatment in appropriate concentrations could remarkably reduce the heart/weight ratio in a concentration-dependent manner (all *P* < 0.05; Figure 1B). The results of HE staining are shown in Figure 1C. Sham-operated mice elicited neatly arranged myocardial fibers with clear structure and complete shape. The myocardial fibers in MI and in TMZ-L-treated mice were disordered, where the cardiomyocytes manifested slight degeneration and severe fibrosis and inflammatory cell infiltration and enlarged spaces were evident. Infiltration and fibrosis in TMZ-M- and TMZ-H-treated mice were minor compared with MI mice, and the lesion could be methodically alleviated with increased TMZ concentration.

Transferase-mediated biotin-16-dUTP staining illustrated a significantly elevated apoptosis rate in MI mice compared to the sham group, while TMZ treatment significantly decreased the myocardial apoptosis induced by MI, with a reducing apoptotic rate with the increase of the TMZ concentration (all *P* < 0.05; Figure 1D). The expression patterns of Bax, Bcl-2, and cleaved caspase-3 were determined by Western blot. Apparent increases in the levels of Bax and cleaved caspase-3 with a decrease in the Bcl-2 level were detected in MI mice. TMZ treatment markedly upregulated the Bcl-2 and downregulated the Bax and cleaved caspase-3 expression patterns in a concentration-dependent manner (all *P* < 0.05; Figure 1E). The aforementioned results indicated that TMZ inhibited MI-induced myocardial apoptosis.

### Trimetazidine Inhibited MI-Induced MEM Disorder

**FIGURE 2:**
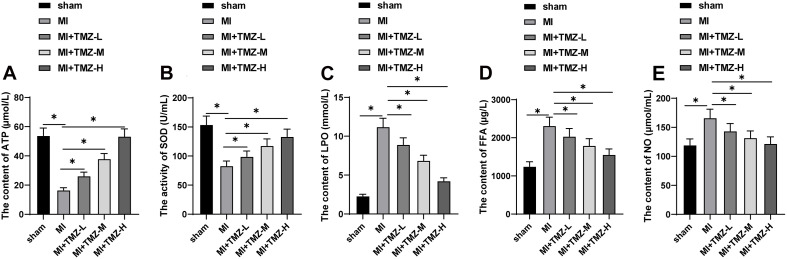
Trimetazidine inhibited MI-induced myocardial energy metabolism disorder. **(A)** ATP content, **(B)** SOD activity, **(C)** LPO content, **(D)** FFA content, and **(E)** NO content in the myocardium (*N* = 10 per group). All data were expressed as the mean ± standard deviation. Data were analyzed using one-way ANOVA, followed by Tukey’s multiple comparisons test. *P < 0.05. ATP, adenosine triphosphate; SOD, superoxide dismutase; LPO, lipid peroxide; FFA, free fatty acids; NO, nitric oxide.

To further explore the effect of TMZ on MEM, we detected the SOD activity and the contents of ATP, LPO, FFA, and NO in myocardial tissues. As shown in Figure 2, the ATP content and SOD activity were significantly reduced, while the contents of LPO, FFA, and NO were considerably upregulated. TMZ treatment attenuated the reduction of ATP content and SOD activity induced by MI and downregulated the contents of LPO, FFA, and NO. The regulatory functions were characterized as TMZ concentration-dependent.

### Trimetazidine Inhibited H_2_O_2_-Induced Myocardial Apoptosis and MEM Disorder

**FIGURE 3:**
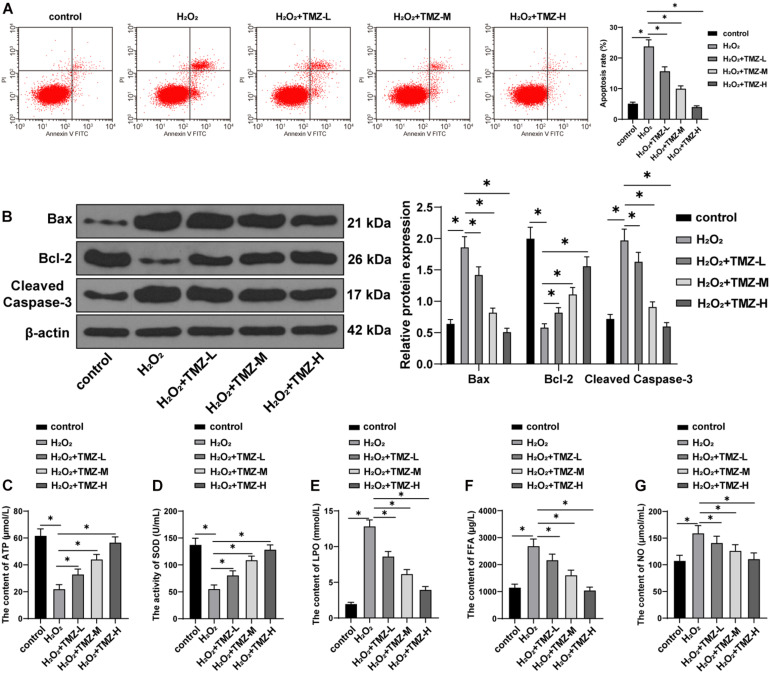
Trimetazidine inhibited H_2_O_2_-induced cardiomyocyte apoptosis and energy metabolism disorder. **(A)** Flow cytometry analysis for apoptosis. **(B)** Western blot analysis of Bax, Bcl-2, and cleaved caspase-3 expressions. **(C)** ATP content in H9C2 cardiomyocytes. **(D)** SOD activity in H9C2 cardiomyocytes. **(E–G)** Contents of LPO in panel **(E)**, FFA in panel **(F)**, and NO in panel **(G)** in H9C2 cardiomyocytes. Cell experiment was repeated three times. All data were expressed as the mean ± standard deviation. Data were analyzed using one-way ANOVA, followed by Tukey’s multiple comparisons test. *P < 0.05. ATP, adenosine triphosphate; SOD, superoxide dismutase; LPO, lipid peroxide; FFA, free fatty acids; NO, nitric oxide.

Next, we sought to validate the regulation of TMZ on apoptosis and the energy metabolism in H_2_O_2_-induced H9C2 cardiomyocytes. Initially, apoptosis was investigated by flow cytometry, and the result showed that H_2_O_2_ treatment significantly induced apoptosis. But TMZ inhibited the apoptosis induced by H_2_O_2_ in a concentration-dependent manner (all *P* < 0.05; Figure 3A). The expression patterns of Bax, Bcl-2, and cleaved caspase-3 were determined by Western blot. The results demonstrated that H_2_O_2_ treatment increased the expression patterns of Bax and cleaved caspase-3 and inhibited Bcl-2 expression. TMZ treatment considerably downregulated the levels of Bax and cleaved caspase-3 and promoted Bcl-2 expression in a concentration-dependent manner (all *P* < 0.05; Figure 3B).

Further energy metabolism results revealed decreased ATP content and SOD activity in the H_2_O_2_ group (Figures 3C,D), while the contents of LPO, FFA, and NO were all increased (Figures 3E–G). TMZ treatment alleviated the reduction of ATP content and SOD activity induced by H_2_O_2_ (Figures 3C,D) and downregulated the contents of LPO, FFA, and NO (Figures 3E–G). All regulating functions were characterized as TMZ concentration-dependent (all *P* < 0.05). The preceding results demonstrated that TMZ inhibited H_2_O_2_-induced myocardial apoptosis and MEM disorder, and 10 μM TMZ was selected for subsequent experiments.

### Trimetazidine Inhibited H_2_O_2_-Induced Myocardial Apoptosis and MEM Disorder *Via* the SIRT1–AMPK Pathway

**FIGURE 4:**
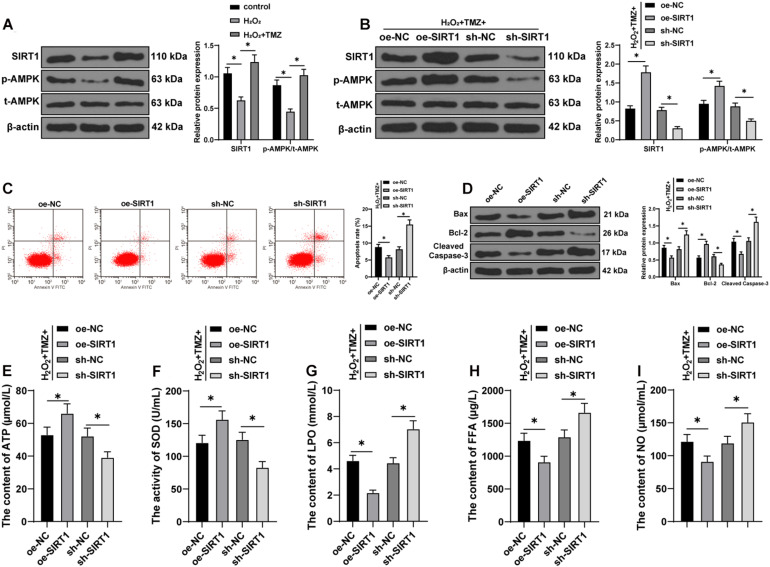
Trimetazidine inhibited H_2_O_2_-induced cardiomyocyte apoptosis and energy metabolism disorder *via* the SIRT1–AMPK pathway. **(A)** Western blot analysis of SIRT1, p-AMPK, and t-AMPK. **(B)** Western blot analysis of SIRT1, p-AMPK, and t-AMPK after overexpressing or silencing SIRT1 in H9C2 cardiomyocytes jointly processed by H_2_O_2_ and TMZ. **(C)** Flow cytometry analysis for apoptosis. **(D)** Western blot analysis of Bax, Bcl-2, and cleaved caspase-3. **(E)** ATP content in H9C2 cardiomyocytes. **(F)** SOD activity in H9C2 cardiomyocytes. **(G–I)** Contents of LPO **(G)**, FFA **(H)**, and NO **(I)** in H9C2 cardiomyocytes. Cell experiment was repeated three times. All data were expressed as the mean ± standard deviation. Data were analyzed using one-way ANOVA, followed by Tukey’s multiple comparisons test. *P < 0.05. ATP, adenosine triphosphate; SOD, superoxide dismutase; LPO, lipid peroxide; FFA, free fatty acids; NO, nitric oxide.

The SIRT1–AMPK pathway is an energy-sensing network with vital involvement in regulating energy metabolism (Tian et al., 2019). Subsequently, the effect of TMZ on the SIRT1–AMPK pathway was investigated in H_2_O_2_-induced H9C2 cardiomyocytes. The results of the Western blot showed that H_2_O_2_ repressed SIRT1 expression and AMPK phosphorylation, while TMZ obtained conflicting results (all *P* < 0.05; Figure 4A), thus indicating that TMZ activated the SIRT1–AMPK pathway.

Furthermore, we overexpressed or silenced SIRT1 expression in H9C2 cardiomyocytes cooperatively processed by H_2_O_2_ and TMZ to validate the function of the SIRT1–AMPK pathway in TMZ regulating H_2_O_2_-induced myocardial apoptosis and MEM disorder. Initially, the SIRT1 expression and AMPK phosphorylation were detected by Western blot, and the results showed that SIRT1 expression and AMPK phosphorylation were increased after SIRT1 overexpression, but decreased after SIRT1 silencing (all *P* < 0.05; Figure 4B). The results of flow cytometry presented a reduced myocardial apoptotic rate after SIRT1 overexpression; however, apoptosis was promoted after SIRT1 silencing (all *P* < 0.05; Figure 4C). Additionally, the overexpression of SIRT1 in H9C2 cardiomyocytes processed by H_2_O_2_ and TMZ inhibited the expressions of Bax and cleaved caspase-3 and promoted Bcl-2 expression, while SIRT1 silencing led to contradictory results (all *P* < 0.05; Figure 4D). All these results suggested that TMZ inhibited H_2_O_2_-induced myocardial apoptosis by the activation of the SIRT1–AMPK pathway.

The results of energy metabolism detection (Figures 4E–I) showed that the overexpression of SIRT1 in H9C2 cardiomyocytes processed by H_2_O_2_ and TMZ increased the ATP content and SOD activity, but decreased the LPO, FFA, and NO contents, while SIRT1 silencing showed conflicting effects (all *P* < 0.05). The results indicated that TMZ could explicitly inhibit the H_2_O_2_-induced MEM disorder by the activation of the SIRT1–AMPK pathway.

### Trimetazidine Inhibited MI-Induced MEM Disorder *Via* the SIRT1–AMPK Pathway *in vivo*

**FIGURE 5:**
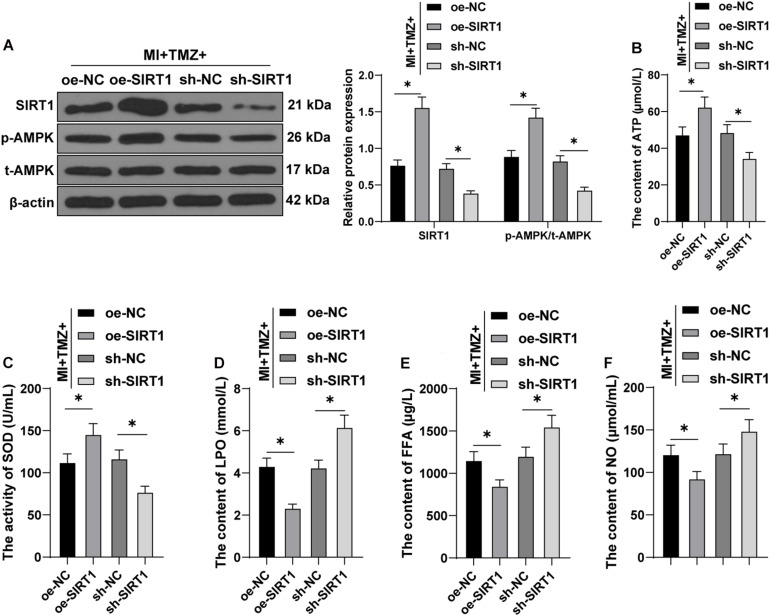
Trimetazidine inhibited MI-induced myocardial energy metabolism *via* the SIRT1–AMPK pathway *in vivo*. **(A)** Western blot analysis of SIRT1, p-AMPK, and t-AMPK. **(B)** ATP content in H9C2 cardiomyocytes. **(C)** SOD activity in H9C2 cardiomyocytes. **(D–F)** Contents of LPO **(D)**, FFA **(E)**, and NO **(F)** in H9C2 cardiomyocytes (*N* = 10 per group). All data were expressed as the mean ± standard deviation. Data were analyzed using one-way ANOVA, followed by Tukey’s multiple comparisons test. *P < 0.05. ATP, adenosine triphosphate; SOD, superoxide dismutase; LPO, lipid peroxide; FFA, free fatty acids; NO, nitric oxide.

We verified that TMZ inhibited H_2_O_2_-induced myocardial apoptosis and energy metabolism disorder by the activation of the SIRT1–AMPK pathway *in vitro*. Further verification was needed *in vivo*. SIRT1 was overexpressed or silenced upon treatment of MI mice with TMZ *in vivo*. SIRT1 expression and AMPK phosphorylation were detected by Western blot. The results showed that the SIRT1 expression and AMPK phosphorylation were increased after overexpressing SIRT1 and decreased after silencing SIRT1 (all *P* < 0.05; Figure 5A). Further detection results of energy metabolism (Figures 5B–F) showed that TMZ treatment and SIRT1 overexpression simultaneously upregulated the ATP content and SOD activity and also downregulated the LPO, FFA, and NO contents. Moreover, TMZ treatment and SIRT1 silencing conjointly reduced the ATP content and SOD activity and increased the LPO, FFA, and NO contents (all *P* < 0.05). The results indicated that TMZ inhibited the MI-induced MEM disorder *via* the SIRT1–AMPK pathway *in vivo*.

### Trimetazidine Inhibited MI-Induced Myocardial Apoptosis *Via* the SIRT1–AMPK Pathway *in vivo*

**FIGURE 6:**
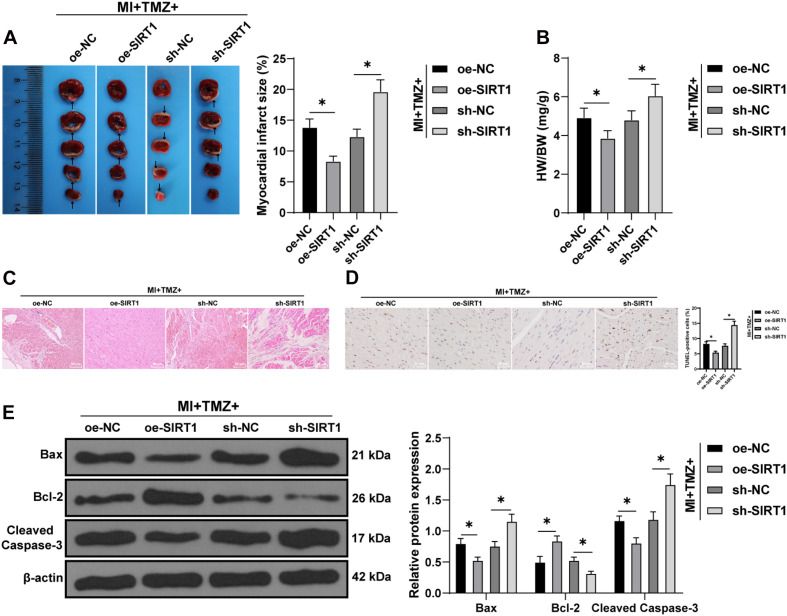
Trimetazidine inhibited MI-induced apoptosis *via* the SIRT1–AMPK pathway *in vivo*. **(A)** MI was detected by TTC staining. *Areas in white* represent the MI region. **(B)** Heart weight was measured and the heart/weight ratio calculated. **(C)** Pathological changes of the myocardial tissue were detected by HE staining. **(D)** Myocardial apoptosis was detected by TUNEL staining. **(E)** Western blot analysis of Bax, Bcl-2, and cleaved caspase-3 (*N* = 10 per group). All data were expressed as the mean ± standard deviation. Data were analyzed using one-way ANOVA, followed by Tukey’s multiple comparisons test. *P < 0.05.

Finally, we tested the action of TMZ on MI-induced myocardial apoptosis *via* regulation of the SIRT1–AMPK pathway *in vivo*. Ultrasonic electrocardiography showed that combination treatment with TMZ and SIRT1 overexpression improved cardiac function, while the silencing of SIRT1 impaired cardiac function in MI mice (all *P* < 0.05; Supplementary Table 2). The TTC results revealed a reduced infarct size in MI mice treated with TMZ and SIRT1 overexpression simultaneously, while SIRT1 silencing increased the infarct size (all *P* < 0.05; Figure 6A). Meanwhile, decreased heart/weight ratios were evident in MI mice treated with TMZ and SIRT1 overexpression; however, SIRT1 silencing increased the heart/weight ratio (all *P* < 0.05; Figure 6B). The results of HE staining are illustrated in Figure 6C. The myocardial fibers were neatly arranged with clear structure and complete shape in mice treated with TMZ and SIRT1 overexpression. However, in mice treated with TMZ and SIRT1 silencing, the myocardial fibers were disorderly with slight cardiomyocyte degeneration and severe fibrosis, with evident inflammatory cell infiltration and enlarged spaces. The lesion of mice in the MI + TMZ + oe-NC and MI + TMZ + sh-NC groups were relatively relieved. TUNEL staining showed lowered myocardial apoptotic rates of MI mice treated with TMZ and SIRT1 overexpression, but the combination of TMZ and SIRT1 silencing promoted apoptosis (Figure 6D). The expressions of Bax and cleaved caspase-3 were decreased while Bcl-2 expression was increased after treatment with TMZ and SIRT1 overexpression. On the contrary, the expressions of Bax and cleaved caspase-3 were increased while Bcl-2 expression was decreased after treatment with TMZ and SIRT1 silencing (all *P* < 0.05; Figure 6E). These results demonstrated that TMZ inhibited MI-induced myocardial apoptosis *via* the SIRT1–AMPK pathway *in vivo*.

## Discussion

The mortality rate associated with MI has been alarmingly high in recent years (Xie et al., 2020). A recent finding has highlighted the ability of TMZ to prevent cardiac rupture, which is an appalling complication of MI (Gong et al., 2018). It is significant to explore effective treatment protocols for MI with TMZ as an advancement. This study explored the effect of TMZ on MI-induced MEM disorder *via* the SIRT1–AMPK pathway and its functional mechanism, which provided a theoretical basis and research insight to identifying a target of MI prevention.

Trimetazidine, depending on the type of disease, may exhibit therapeutic or preventive properties in the management of several diseases such as MI and depression (Liu et al., 2018). Clinically, apoptosis is regarded as an early and predominant form of cell death in MI (Senturk et al., 2014). Our findings revealed that TMZ had significantly reduced the infarct size, heart/weight ratio, and the degree of myocardial apoptosis induced by MI in a dose-dependent manner. Yang et al. have revealed the functionality of TMZ pretreatment in significantly inhibiting myocardial apoptosis and improving cardiac function (Liu et al., 2015). In our findings, TMZ treatment markedly upregulated Bcl-2 but downregulated Bax and cleaved caspase-3 expressions. A recent study demonstrated that TMZ protection against cardiac ischemia/reperfusion injury is dependent on the regulation of the Bcl-2/Bax ratio as Bax facilitates apoptosis while Bcl-2 is an anti-apoptotic protein (Zhang et al., 2019). Furthermore, the cell injury model was established with H_2_O_2_ treatment to explore the role of TMZ in H_2_O_2_-induced MI *in vitro*. Expectedly, H_2_O_2_ resulted in myocardial apoptosis, where treatment with TMZ could obliterate the effect. Reductions in the expression of the pro-apoptotic protein Bax and cleaved caspase-3 activation, along with an elevated expression of anti-apoptotic Bcl-2, were evident after TMZ treatment, which is consistent with the research of Wei et al. (2015). Comprehensively, TMZ could inhibit MI-induced myocardial apoptosis.

Abnormalities of MEM appear as common underlying ailments in cardiac disorders (Marzilli et al., 2019). The optimizing effect of TMZ has been shown in MEM (Yang et al., 2019). The ATP ratio is an index for the determination of energy metabolism in the myocardium (Chen et al., 2016). Increased myocardial LPO and FFA suggest irreversible myocardial damage, while a decrease in SOD activity may be subsequent for free radical-induced myocardial damage (Haleagrahara et al., 2011; Roy et al., 2013). High levels of NO can induce apoptosis and exacerbate existing cardiac dysfunction (Jackson et al., 2008). In our findings, it was evident that the reductions in ATP content and SOD activity induced by MI were attenuated by TMZ treatment, while the contents of LPO, FFA, and NO were downregulated. An existing study has discovered the potential of TMZ to partially improve the alterations in rat myocardial metabolism by regulating cardiac metabolic substrates (Zhang et al., 2019). These results showed that TMZ could essentially inhibit MI-induced MEM disorder. In H_2_O_2_-induced MI *in vitro*, TMZ also inhibited energy metabolism disorders.

The AMPK–SIRT1 signaling pathway is vital for improving mitochondrial energy metabolism (Tian et al., 2019). The AMPK/SIRT1/PGC-1α pathway, by participating in the alleviation of the levels of oxidative stress and apoptosis following MI (Wu et al., 2020), can protect the mitochondrial biogenesis and functions in cerebral ischemic stroke (Gao et al., 2020). In the H_2_O_2_ model, TMZ activated the SIRT1–AMPK pathway. SIRT1 overexpression not only inhibited the expressions of Bax and cleaved caspase-3 and promoted Bcl-2 expression but also elevated the ATP content and SOD activity and reduced the contents of LPO, FFA, and NO in H9C2 cells treated with H_2_O_2_ and TMZ. An existing study has revealed that TMZ decreases the myocardial infarct size and shifts metabolism through regulation of the AMPK and ERK signaling pathways (Liu et al., 2016). The AMPK/SIRT1/PGC-1α pathway is involved in the regulation of energy metabolism in an ischemic heart rat model (Meng et al., 2019). Existing results have suggested that SIRT1 inhibits apoptosis from hypoxic stress and diabetic cardiomyopathy (Guo et al., 2015; Luo et al., 2019). The *in vivo* results were consistent with the *in vitro* results. Conclusively, TMZ inhibited MI-induced myocardial apoptosis and MEM disorder *via* the SIRT1–AMPK pathway.

## Conclusion

In conclusion, TMZ, *via* the activation of the SIRT1–AMPK pathway, could radically inhibit MI-induced myocardial apoptosis and MEM disorder. However, the underlying mechanism and the relation of TMZ in the SIRT1–AMPK pathway have not been methodically explored. Furthermore, elucidating the specific regulatory mechanisms of TMZ in SIRT1 expression and posttranslational modification of the SIRT1-related proteins in MI is essential in future studies.

## Data Availability Statement

The original contributions presented in the study are included in the article/Supplementary Material, further inquiries can be directed to the corresponding author.

## Ethics Statement

The animal study was reviewed and approved by all animal experiments were carried out on the basis of the guidelines for experimental animal care and use, and were approved by the Animal Care and Use Committee of Second Affiliated Hospital of Zhejiang University.

## Author Contributions

X-YL conceptualized the study, contributed to the methodology, and funding acquisition. ZZ curated the data and wrote the original draft. A-GC helped with visualization and investigation. W-WZ supervised the study. X-DW wrote, reviewed, and edited the manuscript. All authors contributed to the article and approved the submitted version.

## Conflict of Interest

The authors declare that the research was conducted in the absence of any commercial or financial relationships that could be construed as a potential conflict of interest.

## References

[B1] Amoedo N. D. Sarlak S. Obre E. Esteves P. Begueret H. Kieffer Y. (2021). Targeting the mitochondrial trifunctional protein restrains tumor growth in oxidative lung carcinomas. *J. Clin. Invest.* 131:e133081. 10.1172/JCI133081 33393495 PMC7773363

[B2] Bajaj A. Sethi A. Rathor P. Suppogu N. Sethi A. (2015). Acute complications of myocardial infarction in the current era: diagnosis and management. *J. Investig. Med.* 63 844–855. 10.1097/JIM.0000000000000232 26295381

[B3] Chen A. Li W. Chen X. Shen Y. Dai W. Dong Q. (2016). Trimetazidine attenuates pressure overload-induced early cardiac energy dysfunction via regulation of neuropeptide Y system in a rat model of abdominal aortic constriction. *BMC Cardiovasc. Disord.* 16:225. 10.1186/s12872-016-0399-8 27855650 PMC5112876

[B4] Chen J. Lai J. Yang L. Ruan G. Chaugai S. Ning Q. (2016). Trimetazidine prevents macrophage-mediated septic myocardial dysfunction via activation of the histone deacetylase sirtuin 1. *Br. J. Pharmacol.* 173 545–561. 10.1111/bph.13386 26566260 PMC4728416

[B5] Chen S. Yang B. Xu Y. Rong Y. Qiu Y. (2018). Protection of luteolin-7-O-glucoside against apoptosis induced by hypoxia/reoxygenation through the MAPK pathways in H9c2 cells. *Mol. Med. Rep.* 17 7156–7162. 10.3892/mmr.2018.8774 29568918 PMC5928668

[B6] Ferrari R. Ford I. Fox K. Challeton J. P. Correges A. Tendera M. (2020). Efficacy and safety of trimetazidine after percutaneous coronary intervention (ATPCI): a randomised, double-blind, placebo-controlled trial. *Lancet* 396 830–838. 10.1016/S0140-6736(20)31790-6 32877651

[B7] Foglio E. Pellegrini L. Germani A. Russo M. A. Limana F. (2019). HMGB1-mediated apoptosis and autophagy in ischemic heart diseases. *Vasc. Biol.* 1 H89–H96. 10.1530/VB-19-0013 32923959 PMC7439920

[B8] Fulco M. Sartorelli V. (2008). Comparing and contrasting the roles of AMPK and SIRT1 in metabolic tissues. *Cell Cycle* 7 3669–3679. 10.4161/cc.7.23.7164 19029811 PMC2607479

[B9] Gao J. Qian T. Wang W. (2020). CTRP3 activates the AMPK/SIRT1-PGC-1alpha pathway to protect mitochondrial biogenesis and functions in cerebral ischemic stroke. *Neurochem. Res.* 45 3045–3058. 10.1007/s11064-020-03152-6 33098065

[B10] Gong W. Ma Y. Li A. Shi H. Nie S. (2018). Trimetazidine suppresses oxidative stress, inhibits MMP-2 and MMP-9 expression, and prevents cardiac rupture in mice with myocardial infarction. *Cardiovasc. Ther.* 36:e12460. 10.1111/1755-5922.12460 30019466

[B11] Guo R. Liu W. Liu B. Zhang B. Li W. Xu Y. (2015). SIRT1 suppresses cardiomyocyte apoptosis in diabetic cardiomyopathy: an insight into endoplasmic reticulum stress response mechanism. *Int. J. Cardiol.* 191 36–45. 10.1016/j.ijcard.2015.04.245 25965594

[B12] Haleagrahara N. Varkkey J. Chakravarthi S. (2011). Cardioprotective effects of glycyrrhizic acid against isoproterenol-induced myocardial ischemia in rats. *Int. J. Mol. Sci.* 12 7100–7113. 10.3390/ijms12107100 22072938 PMC3211029

[B13] Hattori Y. Ihara M. (2016). [Sirt1]. *Nihon Rinsho.* 74 589–594. 27333745

[B14] Hausenloy D. J. Yellon D. M. (2013). Myocardial ischemia-reperfusion injury: a neglected therapeutic target. *J. Clin. Invest.* 123 92–100. 10.1172/JCI62874 23281415 PMC3533275

[B15] Jackson P. E. Feng Q. P. Jones D. L. (2008). Nitric oxide depresses connexin 43 after myocardial infarction in mice. *Acta Physiol. (Oxf.)* 194 23–33. 10.1111/j.1748-1716.2008.01858.x 18394025

[B16] Jain S. Bharal N. Khurana S. Mediratta P. K. Sharma K. K. (2011). Anticonvulsant and antioxidant actions of trimetazidine in pentylenetetrazole-induced kindling model in mice. *Naunyn Schmiedebergs Arch Pharmacol.* 383 385–392. 10.1007/s00210-011-0606-1 21318336

[B17] Jian J. Xuan F. Qin F. Huang R. (2015). Bauhinia championii flavone inhibits apoptosis and autophagy via the PI3K/Akt pathway in myocardial ischemia/reperfusion injury in rats. *Drug Des. Devel. Ther.* 9 5933–5945. 10.2147/DDDT.S92549 26604691 PMC4642812

[B18] Kallistratos M. S. Poulimenos L. E. Giannitsi S. Tsinivizov P. Manolis A. J. (2019). Trimetazidine in the prevention of tissue ischemic conditions. *Angiology* 70 291–298. 10.1177/0003319718780551 29888611

[B19] Leung K. S. Galano J. M. Oger C. Durand T. Lee J. C. (2021). Enrichment of alpha-linolenic acid in rodent diet reduced oxidative stress and inflammation during myocardial infarction. *Free Radic. Biol. Med.* 162 53–64. 10.1016/j.freeradbiomed.2020.11.025 33271280

[B20] Li H. Ma Z. Zhai Y. Lv C. Yuan P. Zhu F. (2020). Trimetazidine ameliorates myocardial metabolic remodeling in isoproterenol-induced rats through regulating ketone body metabolism via activating AMPK and PPAR alpha. *Front. Pharmacol.* 11:1255. 10.3389/fphar.2020.01255 32922293 PMC7457052

[B21] Lim S. H. Lee J. (2017). Xyloglucan intake attenuates myocardial injury by inhibiting apoptosis and improving energy metabolism in a rat model of myocardial infarction. *Nutr. Res.* 45 19–29. 10.1016/j.nutres.2017.07.003 29037328

[B22] Liu M. Wei W. Stone C. R. Zhang L. Tian G. Ding J. N. (2018). Beneficial effects of trimetazidine on expression of serotonin and serotonin transporter in rats with myocardial infarction and depression. *Neuropsychiatr. Dis. Treat.* 14 787–797. 10.2147/NDT.S157441 29588593 PMC5859911

[B23] Liu Y. C. Li L. Su Q. Liu T. Tang Z. L. (2015). Trimetazidine pretreatment inhibits myocardial apoptosis and improves cardiac function in a Swine model of coronary microembolization. *Cardiology* 130 130–136. 25612843 10.1159/000369246

[B24] Liu Z. Chen J. M. Huang H. Kuznicki M. Zheng S. Sun W. (2016). The protective effect of trimetazidine on myocardial ischemia/reperfusion injury through activating AMPK and ERK signaling pathway. *Metabolism* 65 122–130. 10.1016/j.metabol.2015.10.022 26892523 PMC4967934

[B25] Lopaschuk G. D. Ussher J. R. (2016). Evolving concepts of myocardial energy metabolism: more than just fats and carbohydrates. *Circ. Res.* 119 1173–1176. 10.1161/CIRCRESAHA.116.310078 28051784

[B26] Lu L. Liu M. Sun R. Zheng Y. Zhang P. (2015). Myocardial infarction: symptoms and treatments. *Cell Biochem. Biophys.* 72 865–867. 25638347 10.1007/s12013-015-0553-4

[B27] Luo G. Jian Z. Zhu Y. Zhu Y. Chen B. Ma R. (2019). Sirt1 promotes autophagy and inhibits apoptosis to protect cardiomyocytes from hypoxic stress. *Int. J. Mol. Med.* 43 2033–2043. 10.3892/ijmm.2019.4125 30864731 PMC6443335

[B28] Marzilli M. Vinereanu D. Lopaschuk G. Chen Y. Dalal J. J. Danchin N. (2019). Trimetazidine in cardiovascular medicine. *Int. J. Cardiol.* 293 39–44. 10.1016/j.ijcard.2019.05.063 31178223

[B29] Meng H. Wang Q. Y. Li N. He H. Lu W. J. Wang Q. X. (2019). Danqi tablet () regulates energy metabolism in ischemic heart rat model through AMPK/SIRT1-PGC-1alpha pathway. *Chin. J. Integr. Med.* 10.1007/s11655-019-3040-8 [Epub ahead of print]. 31144160

[B30] Mori J. Zhang L. Oudit G. Y. Lopaschuk G. D. (2013). Impact of the renin-angiotensin system on cardiac energy metabolism in heart failure. *J. Mol. Cell Cardiol.* 63 98–106. 10.1016/j.yjmcc.2013.07.010 23886814

[B31] Qi D. Young L. H. (2015). AMPK: energy sensor and survival mechanism in the ischemic heart. *Trends Endocrinol. Metab.* 26 422–429. 10.1016/j.tem.2015.05.010 26160707 PMC4697457

[B32] Roy V. K. Kumar A. Joshi P. Arora J. Ahanger A. M. (2013). Plasma free fatty acid concentrations as a marker for acute myocardial infarction. *J. Clin. Diagn. Res.* 7 2432–2434. 10.7860/JCDR/2013/7682.3566 24392364 PMC3879868

[B33] Senturk T. Cavun S. Avci B. Yermezler A. Serdar Z. Savci V. (2014). Effective inhibition of cardiomyocyte apoptosis through the combination of trimetazidine and N-acetylcysteine in a rat model of myocardial ischemia and reperfusion injury. *Atherosclerosis* 237 760–766. 10.1016/j.atherosclerosis.2014.10.091 25463117

[B34] Shi C. C. Pan L. Y. Zhao Y. Q. Li Q. Li J. G. (2020). MicroRNA-323-3p inhibits oxidative stress and apoptosis after myocardial infarction by targeting TGF-beta2/JNK pathway. *Eur. Rev. Med. Pharmacol. Sci.* 24 6961–6970. 10.26355/eurrev_202006_21688 32633390

[B35] Shu H. Peng Y. Hang W. Zhou N. Wang D. W. (2020). Trimetazidine in heart failure. *Front. Pharmacol.* 11:569132. 10.3389/fphar.2020.569132 33597865 PMC7883591

[B36] Tan H. Qi J. Fan B. Y. Zhang J. Su F. F. Wang H. T. (2018). MicroRNA-24-3p attenuates myocardial ischemia/reperfusion injury by suppressing RIPK1 expression in mice. *Cell Physiol. Biochem.* 51 46–62. 10.1159/000495161 30439713

[B37] Tao L. Bei Y. Lin S. Zhang H. Zhou Y. Jiang J. (2015). Exercise training protects against acute myocardial infarction via improving myocardial energy metabolism and mitochondrial biogenesis. *Cell Physiol. Biochem.* 37 162–175. 10.1159/000430342 26303678

[B38] Tian L. Cao W. Yue R. Yuan Y. Guo X. Qin D. (2019). Pretreatment with Tilianin improves mitochondrial energy metabolism and oxidative stress in rats with myocardial ischemia/reperfusion injury via AMPK/SIRT1/PGC-1 alpha signaling pathway. *J. Pharmacol. Sci.* 139 352–360. 30910451 10.1016/j.jphs.2019.02.008

[B39] Wei J. Xu H. Shi L. Tong J. Zhang J. (2015). Trimetazidine protects cardiomyocytes against hypoxia-induced injury through ameliorates calcium homeostasis. *Chem. Biol. Interact.* 236 47–56. 10.1016/j.cbi.2015.04.022 25937560

[B40] Wu F. Li Z. Cai M. Xi Y. Xu Z. Zhang Z. (2020). Aerobic exercise alleviates oxidative stress-induced apoptosis in kidneys of myocardial infarction mice by inhibiting ALCAT1 and activating FNDC5/Irisin signaling pathway. *Free Radic. Biol. Med.* 158 171–180. 10.1016/j.freeradbiomed.2020.06.038 32726688

[B41] Xie S. Deng W. Chen J. Wu Q. Q. Li H. Wang J. (2020). Andrographolide protects against adverse cardiac remodeling after myocardial infarction through enhancing Nrf2 signaling pathway. *Int. J. Biol. Sci.* 16 12–26. 10.7150/ijbs.37269 31892842 PMC6930369

[B42] Yan H. Jia S. Mao P. (2020). Melatonin priming alleviates aging-induced germination inhibition by regulating beta-oxidation, protein translation, and antioxidant metabolism in oat (Avena sativa L.) seeds. *Int. J. Mol. Sci.* 21:1898. 10.3390/ijms21051898 32164355 PMC7084597

[B43] Yang J. Zhang L. Liu C. Zhang J. Yu S. Yu J. (2019). Trimetazidine attenuates high-altitude fatigue and cardiorespiratory fitness impairment: a randomized double-blinded placebo-controlled clinical trial. *Biomed. Pharmacother.* 116:109003. 10.1016/j.biopha.2019.109003 31125823

[B44] Yang Y. Li N. Chen T. Zhang C. Liu L. Qi Y. (2019). Trimetazidine ameliorates sunitinib-induced cardiotoxicity in mice via the AMPK/mTOR/autophagy pathway. *Pharm. Biol.* 57 625–631. 31545912 10.1080/13880209.2019.1657905 PMC6764339

[B45] Yao Y. Yang L. Feng L. F. Yue Z. W. Zhao N. H. Li Z. (2020). IGF-1C domain-modified hydrogel enhanced the efficacy of stem cells in the treatment of AMI. *Stem Cell Res. Ther.* 11:136. 10.1186/s13287-020-01637-3 32216819 PMC7098145

[B46] Zhang H. Liu M. Zhang Y. Li X. (2019). Trimetazidine attenuates exhaustive exercise-induced myocardial injury in rats via regulation of the Nrf2/NF-kappaB signaling pathway. *Front. Pharmacol.* 10:175. 10.3389/fphar.2019.00175 30890937 PMC6411712

[B47] Zhang X. Liu C. Liu C. Wang Y. Zhang W. Xing Y. (2019). Trimetazidine and lcarnitine prevent heart aging and cardiac metabolic impairment in rats via regulating cardiac metabolic substrates. *Exp. Gerontol.* 119 120–127. 10.1016/j.exger.2018.12.019 30639303

[B48] Zhang X. Y. Wang L. Yan W. J. Lu X. T. Li X. Y. Sun Y. Y. (2020). Period 2-induced activation of autophagy improves cardiac remodeling after myocardial infarction. *Hum. Gene Ther.* 31 119–128. 10.1089/hum.2019.146 31822134

[B49] Zhang Y. Li C. Li X. Wu C. Zhou H. Lu S. (2020). Trimetazidine improves hepatic lipogenesis and steatosis in nonalcoholic fatty liver disease via AMPKChREBP pathway. *Mol. Med. Rep.* 22 2174–2182. 10.3892/mmr.2020.11309 32705195 PMC7411376

[B50] Zhu K. Zheng Y. S. Fang Y. (2020). Effect of trimetazidine on incidence of major adverse cardiac events in coronary artery disease patients undergoing percutaneous coronary intervention: a protocol for systematic review and meta-analysis. *Medicine (Baltimore)* 99:e22918. 10.1097/MD.0000000000022918 33126352 PMC7598800

