# Supplementary material for: Function and Mechanism of Trimetazidine in Myocardial Infarction-Induced Myocardial Energy Metabolism Disorder Through the SIRT1–AMPK Pathway
Source: Front Physiol. 2021 Jun 17;12:645041. doi: 10.3389/fphys.2021.645041 (PMC8248253; doi:10.3389/fphys.2021.645041)
Supplement: Supplementary file 1 [file Data_Sheet_1.docx]

**Supplementary Table 1** Ultrasonic electrocardiography ($\overline{x\text{ }}\text{±s}$)

| Groups/parameters | LVEF(%) | LVFS(%) | LVIDd(mm) | LVIDs(mm) |
| --- | --- | --- | --- | --- |
| Sham group | 60.14±8.07 | 60.16±3.34 | 5.01±0.42 | 4.01±0.42 |
| MI group | 34.16±3.62* | 20.15±3.64* | 8.46±0.84* | 7.46±0.84* |
| MI+TMZ-L group | 44.16±3.62* | 32.34±2.04* | 7.46±0.84* | 6.46±0.84* |
| MI+TMZ-M group | 51.16±3.62* | 39.34±2.04* | 7.01±0.84* | 6.01±0.84* |
| MI+TMZ-H group | 56.34±7.84* | 46.34±2.04* | 6.51±0.84* | 5.21±0.84* |

Note: MI, myocardial infarction; TMZ, trimetazidine; L, low; M, medium; H, high; LVEF, left ventricular ejection fraction; LVFS, left ventricular fraction shortening; LVIDd, left ventricular internal diameter at end-diastole; LVIDs, Left ventricular internal diameter at end-systole.

**Supplementary Table 2** Ultrasonic electrocardiography ($\overline{x\text{ }}\text{±s}$)

| Groups/parameters | LVEF(%) | LVFS(%) | LVIDd(mm) | LVIDs(mm) |
| --- | --- | --- | --- | --- |
| MI+TMZ+oe-NC group | 34.16±3.62* | 21.15±3.64* | 8.36±0.84* | 7.16±0.84* |
| MI+TMZ+oe- SIRT1 group | 54.34±7.84* | 46.34±2.04* | 6.31±0.84* | 5.41±0.84* |
| MI+TMZ+sh-NC group | 36.16±3.62* | 22.15±3.64* | 8.26±0.84* | 7.36±0.84* |
| MI+TMZ+sh-SIRT1 group | 21.34±7.84* | 17.34±2.04* | 9.51±0.84* | 9.21±0.84* |

Note: MI, myocardial infarction; TMZ, trimetazidine; SIRT1, silence information regulator 1; LVEF, left ventricular ejection fraction; LVFS, left ventricular fraction shortening; LVIDd, left ventricular internal diameter at end-diastole; LVIDs, Left ventricular internal diameter at end-systole.
